# Socioeconomic Variations in the Frequency of Parent Number Talk: A Meta-Analysis

**DOI:** 10.3390/educsci12050312

**Published:** 2022-04-29

**Authors:** Eric Dearing, Beth Casey, Pamela E. Davis-Kean, Sarah Eason, Elizabeth Gunderson, Susan C. Levine, Elida V. Laski, Melissa Libertus, Linxi Lu, Caitlin McPherran Lombardi, Ariadne Nelson, Geetha Ramani, María Inés Susperreguy

**Affiliations:** 1 Applied Developmental Psychology, Lynch School of Education and Human Development, Boston College, Chestnut Hill, MA 02467, USA;; 2 Counseling, Developmental, and Educational Psychology Department, Boston College, Chestnut Hill, MA 02467, USA;; 3 Department of Psychology, University of Michigan, Ann Arbor, MI 48109, USA;; 4 Human Development & Family Studies, Purdue University, West Lafayette, IN 47906, USA;; 5 College of Liberal Arts, Temple University, Philadelphia, PA 19072, USA;; 6 Department of Psychology and Comparative Human Development, University of Chicago, Chicago, IL 60637, USA;; 7 Learning Research & Development Center, University of Pittsburgh, Pittsburgh, PA 15260, USA;; 8 Morrissey College of Arts and Sciences, Boston College, Chestnut Hill, MA 02467, USA;; 9 Human Development and Family Sciences, University of Connecticut, Storrs, CT 06269, USA;; 10 Department of Psychology, University of Chicago, Chicago, IL 60637, USA;; 11 Department of Human Development and Quantitative Methodology, University of Maryland, College Park, MD 20742, USA;; 12 Millennium Nucleus for the Study of the Development of Early Math Skills (MEMAT), Pontificia Universidad Católica de Chile, Santiago 7820436, Chile;

**Keywords:** number talk, socioeconomic status, parent talk, child math talk

## Abstract

Using data from 12 studies, we meta-analyze correlations between parent number talk during interactions with their young children (mean sample age ranging from 22 to 79 months) and two aspects of family socioeconomics, parent education, and family income. Potential variations in correlation sizes as a function of study characteristics were explored. Statistically significant positive correlations were found between the amount of number talk in parent-child interactions and both parent education and family income (i.e., r = 0.12 for education and 0.14 for income). Exploratory moderator analyses provided some preliminary evidence that child age, as well as the average level of and variability in socioeconomic status, may moderate effect sizes. The implications of these findings are discussed with special attention to interpreting the practical importance of the effect sizes in light of family strengths and debate surrounding “word gaps”.

## 1. Introduction

There is a large literature demonstrating the importance of parents talking with their young children for brain development, social and cognitive development, language learning, and academic achievement [1–5]. The importance of parent mathematical language appears to be no exception, with correlational and experimental evidence linking parent math talk with young children’s developing math reasoning and skills [6–10]. There is reason to be concerned, however, that socioeconomic (SES) disadvantage may create obstacles for some children and families, limiting opportunities to engage in mathematically enriched conversations in the same way that low SES may limit parent talk, more generally [11].Given that SES-linked variations in math knowledge and skills are evident by school entry, learning opportunities in the early home environment have received increased attention as researchers try to understand the roots of this disparity [12–15].

While controversy surrounds the exact size, nature, and importance of “word gaps” between lower and higher SES families, many studies have reported sizable differences (e.g., *r* of 0.40 and larger) in quantity, variety, and complexity of parent talk as a function of family SES indicators [4,11,16–19]. Yet, little attention has been directed specifically toward SES disparities in parent math talk. In the current study, we conduct a meta-analysis to ascertain a general effect size for the correlation between SES and parental number talk. Specifically, we reviewed data from 12 studies of parent number talk, a domain of parent talk that is robustly predictive of child math skills, but for which the evidence of SES differences is mixed. Our motivation for the study was, in part, to better understand the extent to which number talk might underlie SES variations in child math achievement. While only a direct study of mediators can identify causal mechanisms, larger versus smaller correlations between SES and parent number talk might help direct the future search for such mechanisms.

### 1.1. Variability in Parent Talk by SES

While relatively new in the field of early math learning, the study of children’s exposure to parent talk has a long history in the field of language learning, with now multiple studies and reviews indicating robust relations between language exposure and language growth [5,17,20–23]. It is within this field of study that SES-linked variations in parent talk began to receive attention, with evidence that the quantity and quality of parent talk with young children vary across household factors such as parental education and family income [11,16]. In a recent meta-analysis of 19 studies documenting variations in the quantity of parent speech with infants across low-, middle-, and high-SES families, Dailey and Bergelson [24] report an average effect size for the differences in the quantity of parent talk across these groups, Hedge’s *g*, of 0.41 (*p* < 0.001); when including only studies that were focused on parent speech directed toward the child, the effect size was even larger (i.e., *g* = 0.69, *p* < *0*.001). While these estimates suggest an average “word gap” only about one-third the size that Hart and Risley [20] reported in their seminal study, the meta-analytic difference was still over 500 more words an hour heard by children in the highest-SES families compared with those in the lowest-SES families.

In terms of mechanisms underlying these differences, theory, and research have highlighted the potential roles of (a) human capital (e.g., parent knowledge of child development); (b) constraints on the overall quality of the home environment and its conduciveness to parent-child talk (e.g., learning materials such as books, adequate lighting, room space, family versus work demands, food security [11,25]); (c) the role of economic stress and exposure to chaos for parent psychological well-being (e.g., links between economic stress, parent depressive symptoms, and parent engagement [26,27]); and (d) variations in parent beliefs and attitudes toward child-rearing [24,28].

Yet, mechanisms aside, as Rowe [11] describes in a recent review: There is considerable variability within SES levels, not all areas of parent talk appear to be correlated with SES to the same extent, and the strength of relations between SES and parent talk often differs for sub-components of SES (income and education). Moreover, critiques of the literature point to the limits of narrowly defining the quality of home learning environments solely based on parent speech patterns, and the harms of a deficit-oriented model of families that has emerged among educators due to narratives focused on “word gaps” between households [29–31]. Taken together, it is clear that attention to family strengths, within-group variability, and nuanced accounts of the quality of parent-child interaction beyond parent talk all require greater attention in the field. Yet, robust SES disparities in the quantity of talk in the early home environment are difficult to entirely disregard, and early math researchers should evoke questions regarding the extent to which similar patterns emerge for math talk as for general language exposure.

### 1.2. Parent Number Talk and Children’s Math Learning

Over the last decade, a small (but growing) collection of studies have demonstrated links between parent math talk and young children’s math learning. The frequency with which parents talk about numerical (and spatial) concepts during early childhood has proven to be predictive of both concurrent and later math skills [7,9,10,15,32–34]. Moreover, there is experimental evidence that increasing parent number talk (e.g., by reading number books with their children or directing them to include conversations about math concepts in their play) can improve children’s numerical skills [8,35]. These results appear all the more important when juxtaposed with naturalistic observations indicating tremendous variability in the amount of math talk children hear at home; Levine and colleagues [36] found that while some parents spoke more than 200 number words across five 90-min observations of naturalistic interactions in the home environment when children were between the ages of 14–30 months, others spoke fewer than 10. Very little research, however, has been dedicated to understanding *why* some parents engage in much more number talk (or math talk, more generally) than others (for exceptions, see the work of Elliott and colleagues and the work of Napoli and colleagues [37,38]).

With regard to SES differences in number talk (or other forms of math talk), we are unaware of any studies that have directly addressed this question. Nonetheless, because parent education and family income are common covariates used to rule out selection factors in studies examining number talk and children’s number skills, several studies have reported correlations with these SES indicators [15,32,39,40]. Interestingly, the size of these correlations between number talk and either parent education or family income (or both) have varied considerably, from zero or even negative to positive and moderately large in absolute size (i.e., *r* ≤ 0 to ~0.40).

This variability in correlation size is perhaps not surprising given that most studies of parent math talk are based on small samples. The effort, time, and resources required to conduct observational studies of parent talk (and to transcribe and code this talk in a reliable and thorough manner) have often required researchers to focus their efforts on a limited number of families; many studies in this area have observed 50 or fewer families. The limited power of the individual studies was the impetus for the present study, which provides a meta-analysis of correlations between parent number talk and SES using data from 12 samples.

### 1.3. The Present Study

In the present study, we meta-analyze correlations between parent number talk during interactions with their young children (mean sample age ranging from 22 to 79 months) and two aspects of family socioeconomics, parent education, and family income. Our primary goal was to estimate the average correlation across 12 studies for number talk and parent education as well as number talk and family income. As a secondary goal, we explored potential variations in correlation sizes as a function of study characteristics, including where parent-child interactions were observed (i.e., lab or home), child age, the proportion of sample composed of families of color, study design (i.e., experimental or observational), average education and income level, and variability in education and income level.

## 2. Materials and Methods

The present study is based on secondary (meta-analysis) data originally collected as part of 12 observational or experimental studies of parent-child math talk in early childhood. Studies were identified through Google Scholar keyword searches (i.e., math talk, number talk, early math, parent number talk, maternal number talk, paternal number talk, mother number talk, father number talk, caregiver number talk, early childhood math, parent-child math talk, parent-child number talk, home math talk, home number talk), and emails to authors known to have published on the topic requesting published or unpublished relevant data. Studies published and/or conducted between January 2000 and August 2021 were eligible for inclusion, with earlier dates excluded to minimize historical and demographic changes in family socioeconomics. Studies were included only if published in English. Additional inclusion criteria were: parent number talk data had been collected via direct researcher observation (e.g., video and/or audio recordings); children observed were (on average) no older than eight years of age; if published, the manuscript provided some mention (e.g., in sample description, measures, and/or study results) of either parent education or family income data being collected.

In Tables 1 and 2, we provide an overview of the 12 studies included in our meta-analysis, including the sample sizes, sample demographics, the location of the observations, the type of parent-child learning tasks that were observed, and how parent number talk was operationalized. In total, across these 12 studies, 714 parents were observed interacting with their child with individual study sample sizes ranging from 28 to 119. The average age of children in the studies ranged from 22 to 79 months.

Eleven of the 12 studies were conducted in the U.S., with one study conducted in China. Of those conducted in the U.S., participants were sampled primarily from midwestern and eastern areas of the country near research universities. Among the 11 studies conducted in the U.S., the racial/ethnic composition of the samples differed considerably, including some that were predominantly comprised of families of the color [32,41] and others that were predominantly comprised of White families [15,37]. Of the 12 studies, nine collected SES data on both parent education (mostly maternal education) and family income levels; three studies did not collect family income data.

### 2.1. Procedures

Parent number talk was observed during either unstructured, semi-structured, or structured interactions with children. Studies that examined parent talk during unstructured activities [9,10] were conducted in the home environment during daily routines (e.g., mealtime; play). Semi-structured and structured activities were generally focused on dyadic parent-child play with toys or games, or while engaged in a structured daily routine (e.g., cooking together with researcher-developed recipes and math talk tips). Observed parent-child interactions for studies using semi-structured or structured activities were conducted in a variety of different settings, including the home, in a university laboratory, or in a school or community setting where dyads could be observed in a room together. In four studies [41,44–46], participants were randomly assigned to receive different instructions or interaction materials (i.e., recipes with and without number talk tips, variations in the level of instructional guidance given to parents, variations in board game features, and bounded versus unbounded sets of toys). For the current analyses, correlations between parent number talk and SES were examined across experimental conditions in these four studies.

### 2.2. Measures

#### Parent educational attainment.

Educational attainment at the time of observation was obtained via self-report questionnaires in all studies for the parent interacting with the child. In the majority of studies, the participating parent was the mother. In two studies, either the mother or father engaged in the interaction and that parent’s education level was collected [44,45]. Although the number of response categories (and method of clustering similar levels of education such as technical college and associate’s degrees) differed across studies, all responses were converted to the number of years of education (e.g., high school diploma was converted to 12 years of education). For studies using a single category for participants with less than a high school diploma, this category was assigned 10 years of education. The mean level of education in samples ranged from approximately one year of higher education (i.e., 13.15 years in Ramani et al. [32]) to more than a BA degree (i.e., 17.4 in [44]). The variability in education levels also differed considerably across studies with standard deviations ranging from less than a year (i.e., 0.81 in Eliott et al. [15]) to more than two and half years (i.e., 2.62 in Lombardi & Dearing [37]).

#### Family income.

Family income data were collected via parent self-report questionnaires in nine of the 12 studies. In these nine studies, participants were asked to report their total annual family income. For studies using response categories that included a range of income levels (e.g., $50,001 to $60,000), the range mid-points were used. For the study conducted in China, income was converted to 2020 U.S. dollars. The average income in the studies ranged from less than half the national median income in the year the data were collected (i.e., $26,875, in Ramani et al. [15]) to more than twice that threshold (i.e., $127,636 in Casey et al. [39]). Moreover, within-study variability in income differed markedly across the samples, with standard deviations ranging from less than $15,000 [15] to nearly $70,000 [39].

#### Quantity of parent number talk.

Parent number talk data were collected using either videotaped or audiotaped recordings of parent-child interactions. Transcriptions of audio from these recordings were transcribed by trained research assistants and/or the study authors. Inter-rater reliabilities for coding of number talk across the studies ranged from acceptable (~0.75) to excellent (~0.95).

For the current analyses, the total quantity of number talks parents engaged in with their children was the primary variable of interest when examining correlations between number talk and SES in our meta-analysis. With slight variations, ten of the studies included in the current analyses captured the quantity of number talk with young children across several key domains (e.g., counting, numeral identification, cardinality, ordinal relations, magnitude, and arithmetic). Of the remaining two studies, one study [39] was focused on maternal support of arithmetic including talk about count-on versus count-all strategies, estimation, arithmetic, and math hints or facts (e.g., decomposition strategies); for this study, the total arithmetic talk variable, collapsed across subdomains, was used. The final study [15] was focused primarily on parent talk about counting and labeling set sizes; for these studies, the total number talk in these two domains was used in the current analysis. All of the studies excluded the use of number words that were not used in a numerical sense (e.g., “Do you want to play with this toy or that *one*?”).

#### Moderators.

Several characteristics of the study samples and designs were examined as potential moderators of associations between SES and number talk, including mean parent education, standard deviation for parent education, mean family income, standard deviation for family income, mean child age, proportion of the sample that were families of color, observational versus experimental study (i.e., a dummy variable indicating whether families were randomly assigned to multiple conditions), and where observations were conducted (i.e., a dummy variable indicating if observations were in the home versus in a lab, school, or community setting).

### 2.3. Statistical Analyses

To examine the average effect sizes of associations between SES—parent education and income—and parent number talk, we estimated random-effects meta-analyses using Stata 15 [47–50]. The selection of the random effects (versus fixed effects) was based on our assumption that there is likely distribution of true effect sizes (rather than a single true effect size) for the association between SES and parent number talk, with variation in that distribution potentially a function of factors such as child and family characteristics and the contexts and conditions within which parents and children are interacting (e.g., during family routines versus during more formal math teaching moments). Correlation coefficients, *r*, and the corresponding standard errors from each study were used as the effect sizes of interest.

In addition to average effect sizes, we report *I*^2^, an estimate of the proportion of heterogeneity in effect sizes that were due to variability in the true treatment effect, versus sampling variation. We also conducted exploratory moderator analyses using meta-analytic regression; these analyses were used to explore whether the size of associations between SES and parent number talk covaried with study-specific characteristics of the samples or study designs.

It is important to note that while our meta-analytic estimates of the average association between SES and parent number talk were sufficiently powered, the heterogeneity estimates and moderator analyses were not [51,52]. Meta-analyses (and our analytic strategies) are routinely conducted on study numbers similar to ours (e.g., 10 or fewer studies; [51]), but methodological protections against both Type I and Type II errors are recommended. We obtained chi-square significance tests of the overall level of heterogeneity in effect sizes and *I*^2^, as an estimate of variance due to variability in the true effect, but as recommended by von Hippel [51], we rely on confidence intervals to aid our interpretation of these estimates. Similarly, given the small number of studies, the meta-analytic regression models were exploratory and interpreted with caution; for these models, we focused on effect size (e.g., the size of the association between study characteristics and correlation coefficients) and used a Monte Carlo permutation approach to significance testing that helps protect against Type I errors [52].

## 3. Results

**Figure 1.**
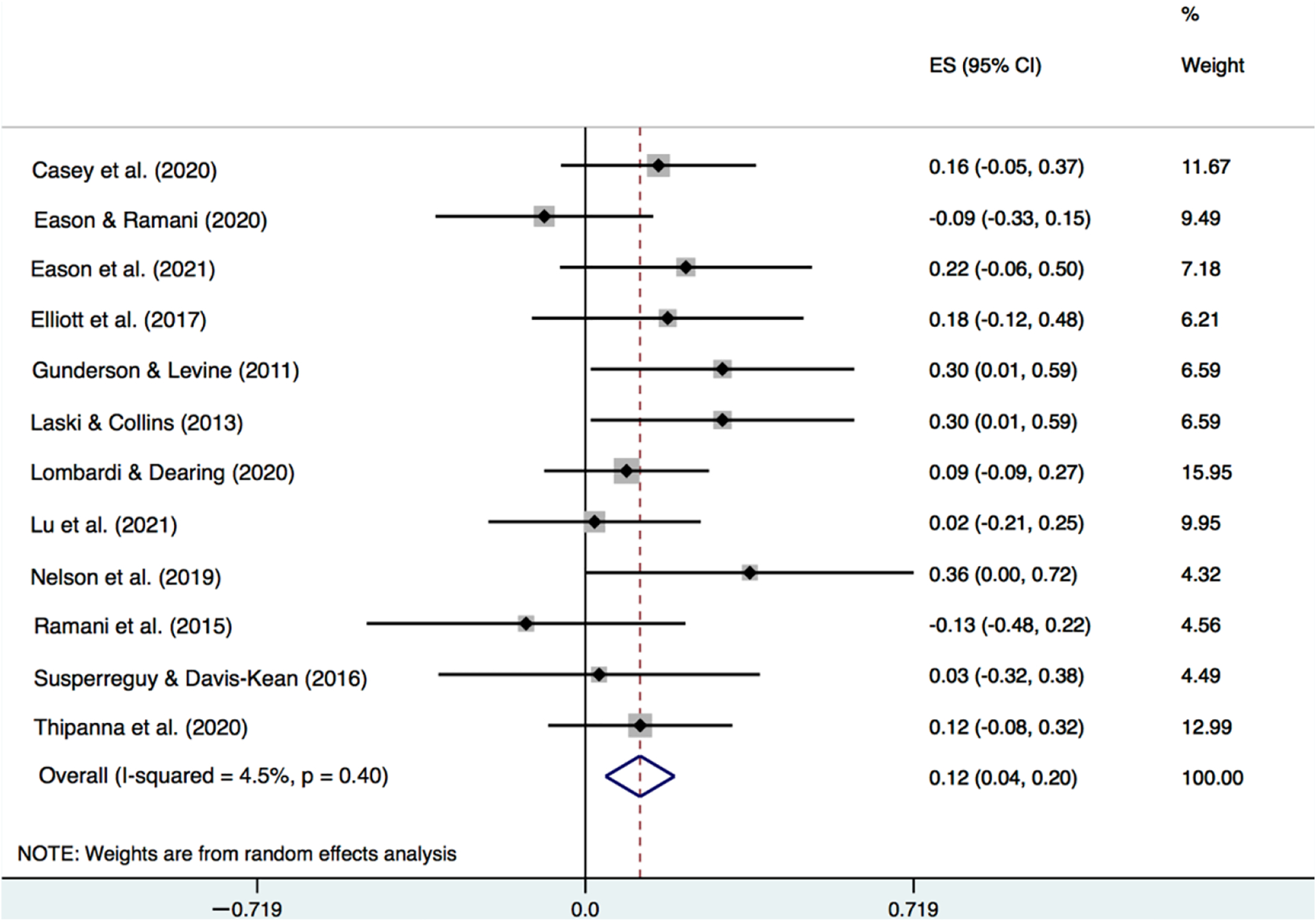
Meta-analytic results for the correlation between parent number talk and parent education [9,10,15,32,33,37,39–41,44–46].

**Figure 2.**
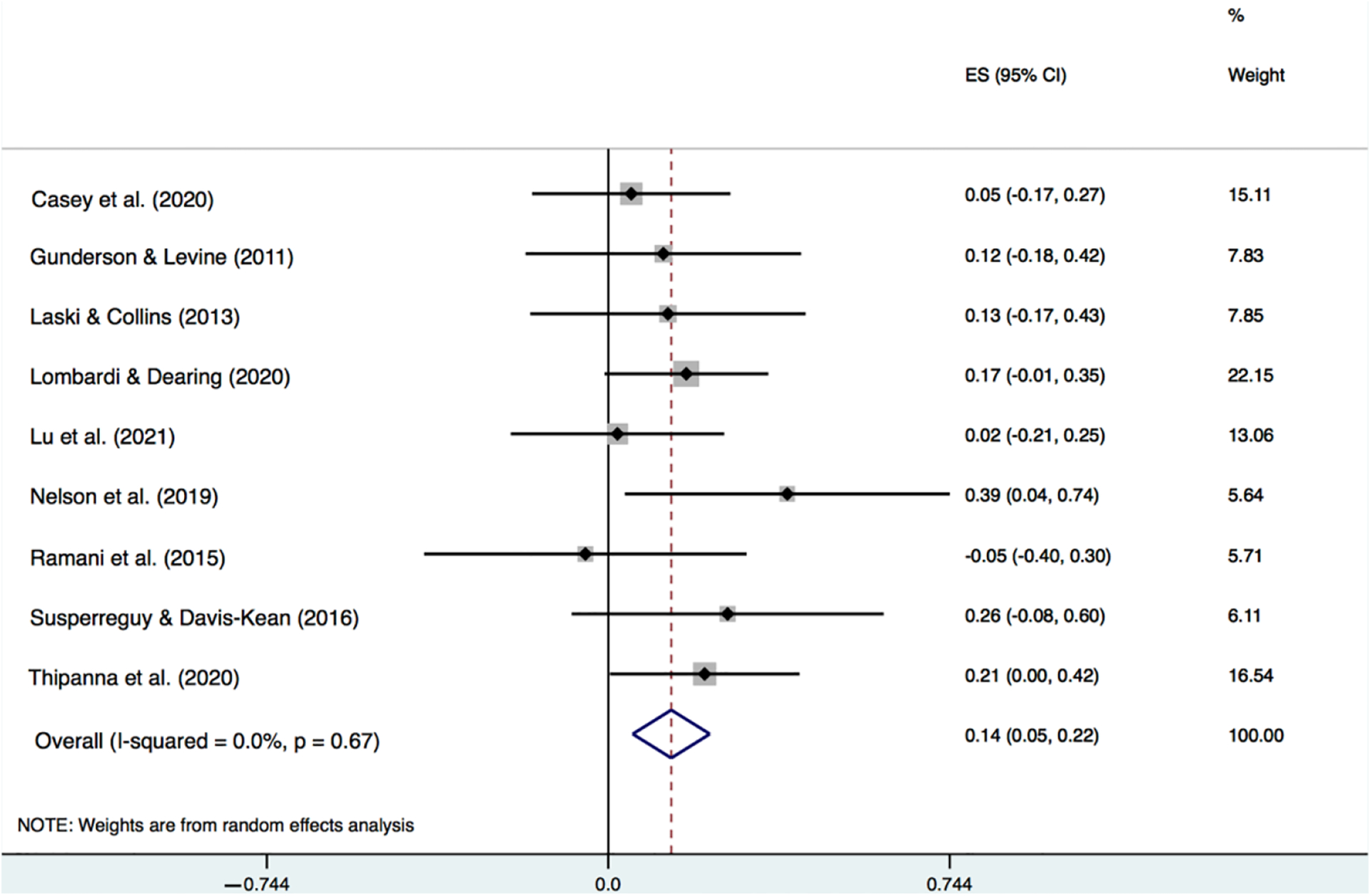
Meta-analytic results for the correlation between parent number talk and family income [9,10,15,32,33,37,39–41,44–46].

In Figure 1, using a forest plot, we report study effect sizes and confidence intervals as well as the pooled meta-analytic effect size and confidence interval for the association between parent education and parent number talk. Additionally reported in Figure 1 is, *I*^2^, the percentage of between-study heterogeneity in this association that was estimated to be due to variability in the true effect size. Similarly, in Figure 2, we report the effect sizes, confidence intervals, and the *I*^2^ statistic for the association between family income and parent number talk.

Focusing first on the results for parent education (Figure 1), the average effect size was positive and statistically significant, although modest, *r* = 0.12, *p* = 0.002. Even at the upper bound of the 95% CI (i.e., 0.04 to 0.19), the true association would not be considered large by conventional standards. It is also worth noting that while correlations in the individual studies varied in size (and direction) from −0.13 to 0.36, the confidence intervals (for all studies) were overlapping, and only 3.9% of the variance was estimated to be due to variability in true effect size. In other words, the preponderance of between-study variance appeared due to sampling.

Turning to the results for family income (Figure 2), the average effect size was also positive and statistically significant, but also modest, *r* = 0.14, *p* = 0.001. As with education, even if the true association between family income and parent number talk was at the upper bound of the 95% CI (i.e., 0.05 to 0.22), it would not be considered large. While the study correlations ranged from −0.05 to 0.39, confidence intervals were overlapping for all studies. In this case, none of the variance was estimated to be due to variability in the true effect.

In attempting to examine the practical significance of the meta-analytic correlations we observed, we also converted the r coefficients into the number of instances that number talk was observed, on average, in homes one standard deviation above and below the mean on education and income. Given that the individual studies had standard deviations ranging from approximately 11 (during a 15-min interaction, [44]) to approximately 44 (averaged across five 90-min interactions, [9]) for frequency of number talk, correlations of 0.14 would translate into differences between low and high SES families of approximately 3 to 12 instances of number talk per researcher-observed interaction, dependent on the length of the interaction as well as variations in contextual affordances and demands.

### 3.1. Exploring Moderators: Meta-Analytic Regression Models

**Figure 3.**
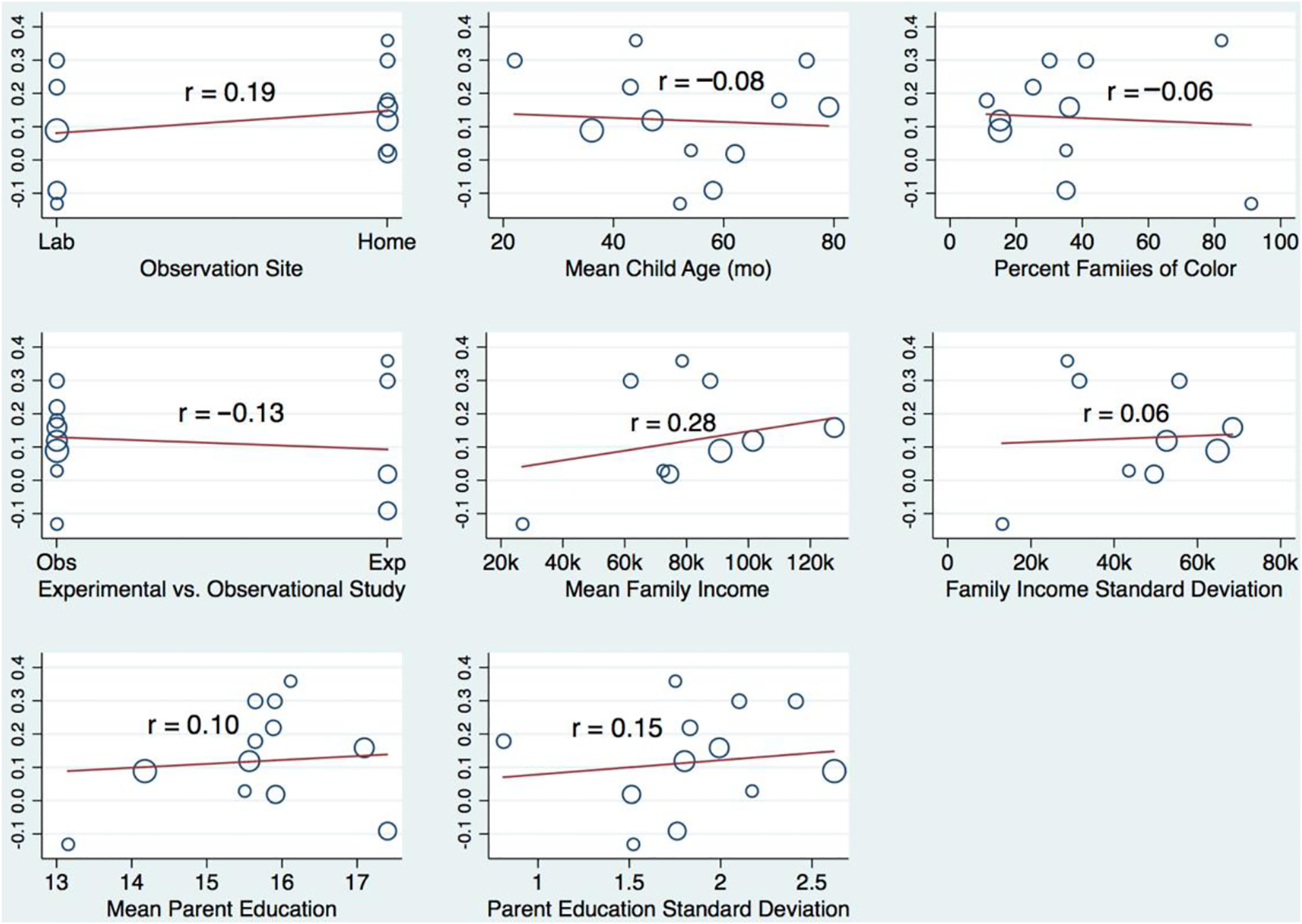
Variation in the correlation between parent education and parent number talk as a function of study characteristics. Vertical axes indicate the size of study correlations with distributions of study characteristics (i.e., potential moderators) indicated along horizontal axes. Study correlation effect size indicators (blue circles) differ in size according to the study sample size. Red lines indicate the linear trend with corresponding r coefficients provided within each graph.

**Figure 4.**
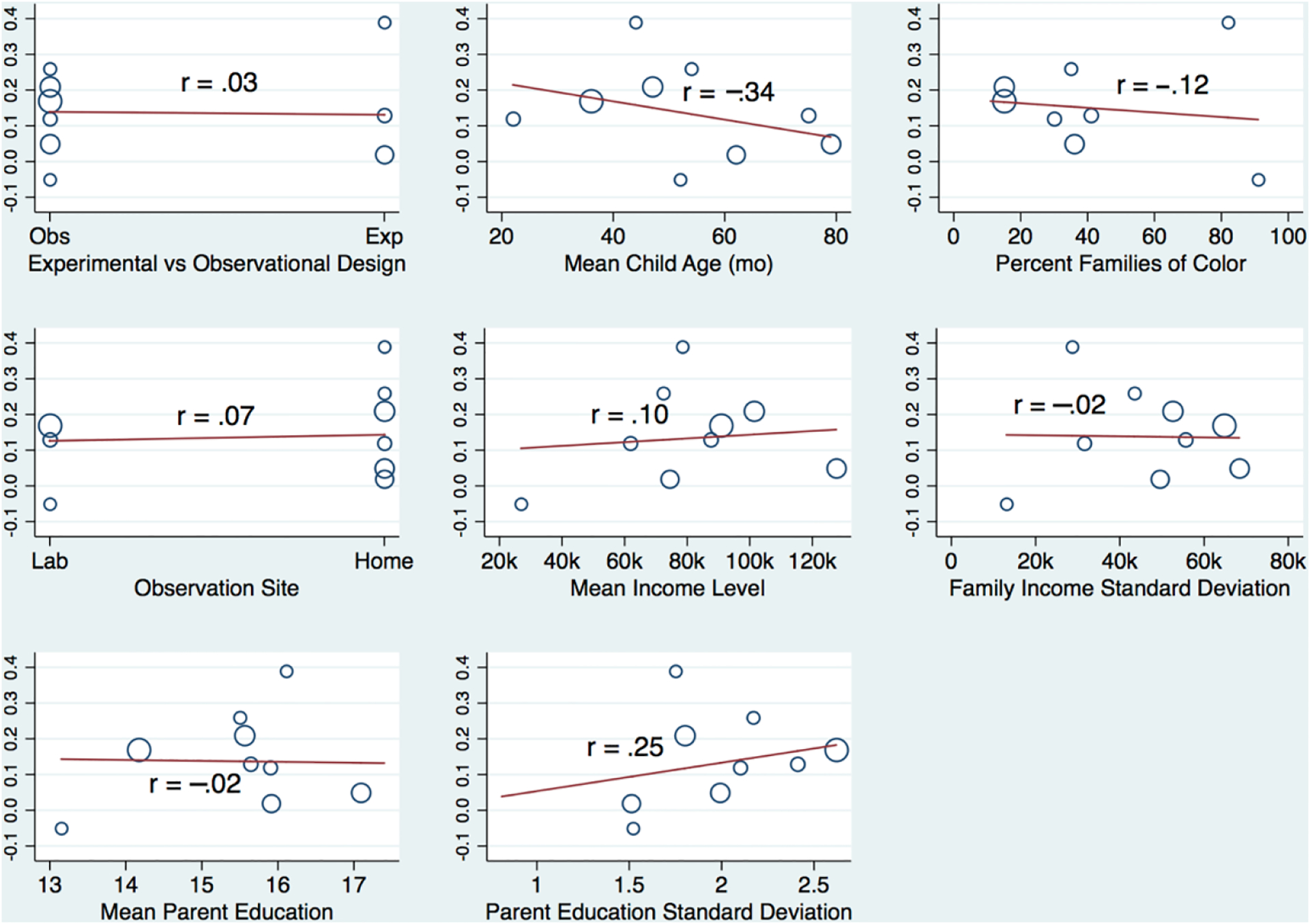
Variation in the correlation between family income and parent number talk as a function of study characteristics. Vertical axes indicate the size of study correlations with distributions of study characteristics (i.e., potential moderators) indicated along horizontal axes. Study correlation effect size indicators (blue circles) differ in size according to the study sample size. Red lines indicate the linear trend with corresponding r coefficients provided within each graph.

As a final step in our analyses, we explored the possibility that effect sizes may vary by sample characteristics and/or study designs. Specifically, we estimated meta-analytic regression models in which effect sizes were regressed on the set of potential moderators. As noted above, given the limited number of studies, power in the meta-analytic regression models was constrained (e.g., for income, only correlations larger than 0.65 would be significant at *p* < 0.05 in the meta-analysis regression models). For this reason, we estimated one potential moderator at a time and focused primarily on effect size for these analyses with all significance tests conducted using a Monte Carlo permutation approach [52]. Graphs of the meta-analytic regression models are displayed in Figure 3 for education and in Figure 4 for income.

Most meta-analytic associations between potential moderators and study effect sizes were close to zero, and none were statistically significant at *p* < 0.05. Indeed, descriptively, only three trends were worth considering (with caveats around their exploratory nature). First, the correlation between parent education and number talk varied somewhat across studies (*r* = 0.28) as a function of average income level in the sample: correlations between education and number talk were somewhat larger in samples that were relatively high with regard to average family income. Second, correlations between family income and number talk varied somewhat (*r* = 0.25) across studies as a function of sample variability in parent education: correlations between income and number talk were somewhat larger in samples with greater variability in parent education. Third, correlations between income and number talk varied somewhat (*r* = −0.34) across studies as a function of average child age in the sample: correlations between income and number talk were somewhat smaller at increasingly older child ages. Again, however, we underscore that these findings should be interpreted with limited power in mind.

### 3.2. Excluding ”Treatment” Conditions

As an extension of our moderator analyses, we also re-estimated the meta-analyses with all participants from the non-experimental studies, but with a more restricted set of participants from the experimental studies [41,44–46]. Specifically, for the experimental studies, we excluded all participants who were in the “treatment” condition(s). This allowed us to focus the meta-analyses more exclusively on participants for whom parent number talk was observed under more naturalistic or “business-as-usual” circumstances. In the Nelson et al. study [41], we included only dyads (*n* = 11) in the control condition (i.e., cookbooks without number talk tips) and excluded dyads who were provided with number talk tips in cookbooks. In the Eason and Ramani study [44], we included only dyads (*n* = 22) in the unguided play condition and excluded dyads in guided play and didactic instruction conditions. In the Lu et al. [45] study, we included only dyads (*n* = 22) who played with unbounded, mixed toy sets and excluded dyads who played with bounded sets of domain-similar toys that were chosen to support number talk. In the Laski et al. study [46], we included only dyads (*n* = 22) who played with a non-linear board game and excluded participants who played with a linear board game designed to support number talk. Compared with our primary analyses that included all participants from these studies, the effect size for the meta-analytic correlation was somewhat larger for parent education (i.e., *r* = 0.16 compared with *r* = 0.12) and slightly smaller for family income (*r* = 0.12 compared with *r* = 0.14) when including only participants assigned to more naturalistic conditions. Yet, the 95% confidence intervals using these two approaches overlapped one another almost entirely, and the meta-analytic effect sizes from these two alternative approaches fell well within each other’s confidence intervals.

### 3.3. Publication Bias

**Figure 5.**
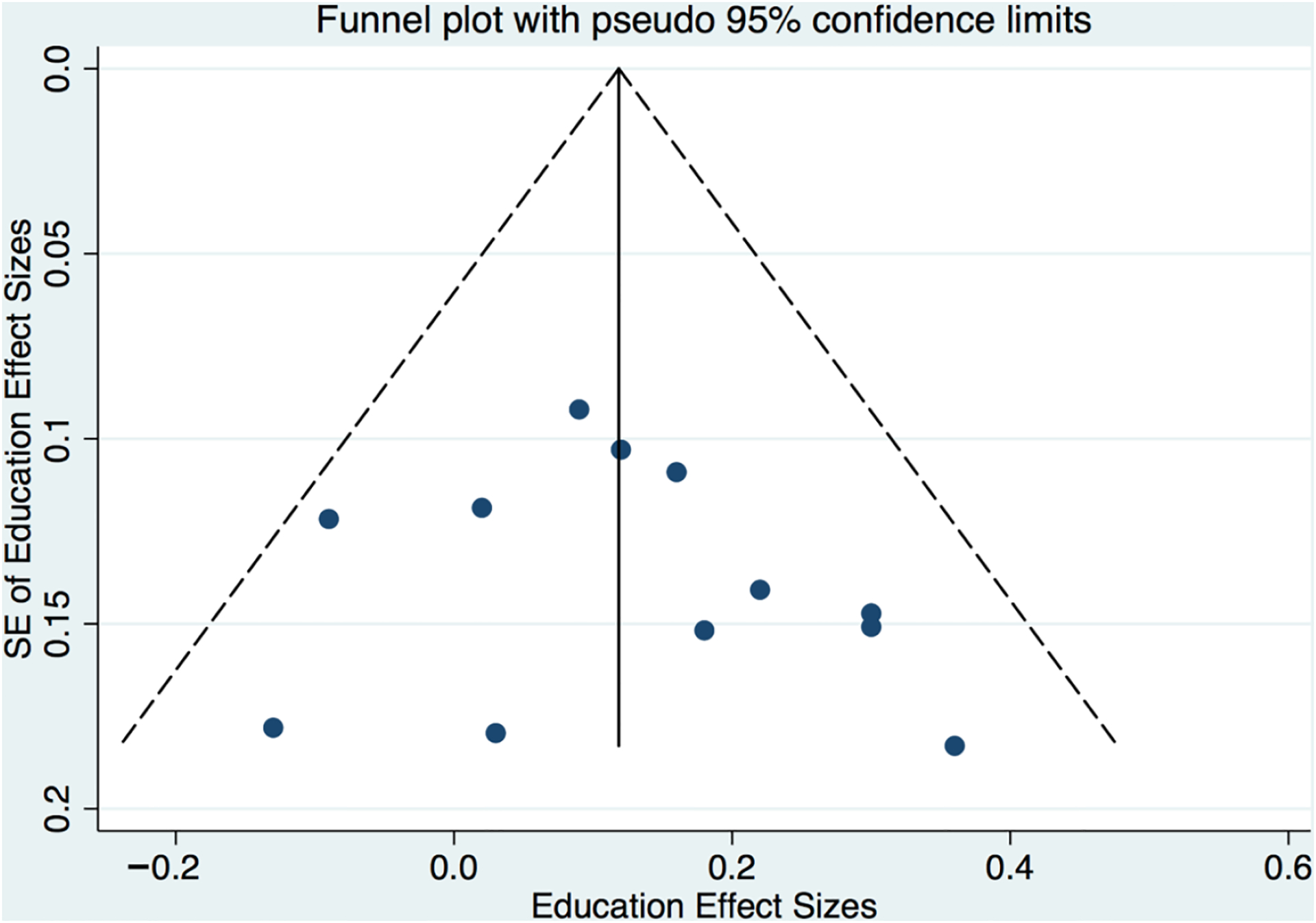
Funnel plot for study correlations between parent education and parent number talk.

**Figure 6.**
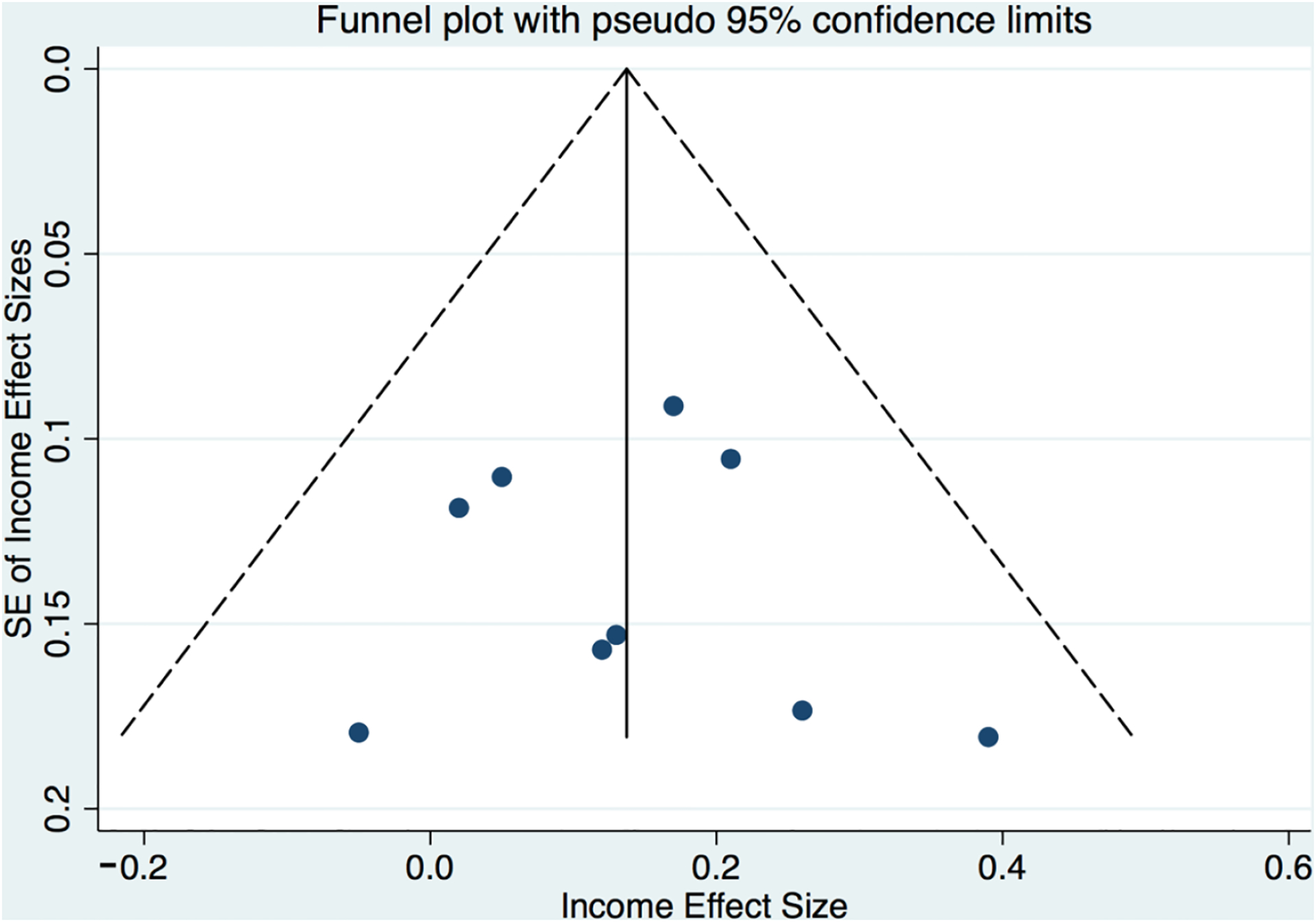
Funnel plot for correlations between family income and parent number talk.

While publication biases (e.g., “file drawer” problems for null studies never published) are ubiquitous concerns in the meta-analysis that are not easily addressed empirically, we found no direct evidence in these data of publication bias using conventional empirical tests. Eggers and Berg tests for small study effects were both null (e.g., Eggers test: *p* = 0.51 for education and *p* = 0.79 for income). In addition, funnel plots (see Figures 5 and 6) also depicted no evidence of publication bias, as standard errors appear uniformly distributed within the plot.

## 4. Discussion

In a meta-analysis of 12 studies, we found statistically significant positive correlations between the amount of number talk in parent-child interactions and both parent education and family income (i.e., *r* = 0.12 for education and 0.14 for income). These results help push the field toward a more synthesized understanding of the extent to which variations in number talk are associated with family SES and, in turn, evoke questions about the extent to which differences in number talk may, or may not, underlie SES disparities in children’s math skills. While the effect sizes for correlations between SES and number talk appear modest at first glance, we discuss several considerations for understanding their practical importance. We argue that these correlations may belie the cumulative importance of enduring exposure to differences of this magnitude across early childhood. We also argue that interpretation of our findings is best done with consideration for factors not captured here such as the quality and diversity of number talk, and qualities of the home learning environment, holistically. We also address the possibility that parent education and family income may not be the most critical factors affecting parent number talk compared with other aspects of family life that intersect with mathematical knowledge and attitudes, a matter worthy of further research.

### 4.1. SES and Parent Number Talk: Average Associations and Practical Significance

Over the last decade, increasing attention has been given to the role of the early home environment in supporting mathematical development [12]. In part, this attention has been driven by evidence of large disparities in early math skills along socioeconomic lines, disparities that appear prior to children entering school [53]. In this line of study, parent-child talk about numbers has emerged as one robust predictor of early math skills, particularly parent talk with young children about key early numerical concepts (e.g., one-to-one correspondence and cardinality). In these studies, however, findings have been quite mixed as to whether number talk varies as a function of SES. Here, in a meta-analysis of 12 studies that captured observational data on parent number talk, we found the average association between the frequency of parent talk about numerical concepts and parent education was 0.12, and it was not much larger for family income at 0.14. While statistically significant in both cases, we focus here on the size of these associations to determine their practical significance (or lack thereof).

As noted by McCartney and Rosenthal [54] p. 175, “There are no easy conventions for determining practical importance. Just as children are best understood in context, so are effect sizes.” This theme has been underscored by others as well with conceptual and empirical recommendations for contextualizing effect sizes, including how the effect size compares with other relevant effect sizes in the field [54], whether the effect size corresponds to a one-time event or an enduring environment [55], how the effect size would translate into more intuitive metrics [56], and the type of research design and overall study quality from which the effect size has been drawn [57]. Here, we follow these lines of advice. We start by juxtaposing the current study effect sizes with meta-analytic effect sizes for SES differences in the amount of total parent talk with children.

Compared with effect sizes in meta-analyses of SES differences in total parent talk (after converting *r* to *g*; [24]), the effect sizes for parent education and family income differences in frequency of parent numerical talk are only about half as large. One possible explanation for this is that the overall amount of talk that parents engage in with their children is more strongly affected by socioeconomic position than is mathematical talk, specifically. We return to this possibility in our discussion below, but first: it is worth considering that parent number talk—compared with all other forms of talk—is exceptionally rare for all families; for example, Eason and Ramani [44] found that of the more than 1300 words that parents spoke, on average, during semi-structured 15-min interactions with their children only 13% of those words involved numerical content (and this dropped to 3% in unguided play). One implication of the general infrequency of number talk is that small differences in frequency may be salient to the child and meaningful in terms of variations in learning opportunities.

It is also critical to note that our effect sizes are based on discrete, often brief, interactions between parents and children. Indeed, in most studies included in our meta-analysis, parents and children were observed just a single time for an interaction lasting as short as a few minutes (for exceptions, see [9,10,37,41]). As Funder and Ozer [55] note, seemingly small effect sizes (e.g., *r* = 0.10) that capture only a snapshot of an ongoing phenomenon can have “hugely important implications” if such phenomena hold the potential to accumulate impact over time. The study of parent number talk in brief, isolated interactions seems a potential case in point. While we estimate that our correlations are indicative of only about 3 to 12 instances of number talk over an isolated researcher-observed interaction, the cumulative exposure to number talk given only three such interactions per week across the first five years of childhood translates into children in higher SES homes hearing parents talk about numbers between 2340 to 9360 more times than children in lower SES homes. It is not unreasonable to speculate that the accumulating, perhaps even compounding, effects of this level of exposure on children’s mathematical learning could be sizable. Yet, this speculation requires assumptions around which the cumulative knowledge is not clear [18]. Despite some of this evidence being captured under naturalistic home conditions [9,10], observations in many studies were conducted under conditions that might not reflect typical family life (e.g., reduced distractions, play materials provided, and the impact of being observed). We do not know how well the amount of parent number talk that occurs during researcher-observed interactions—primarily captured during mother-child dyadic interactions—generalizes to typical day-to-day family life.

That said, even if the developmental consequences of frequency of talk do accumulate over early childhood in important ways, the practical importance of the correlations we report is perhaps most meaningful considered within a broader early learning context. Frequency is only one aspect of parent talk, an aspect that does not capture qualitative factors such as whether and how parents use number talk to initiate, sustain, and contribute to conversations [1,40,58]. Children’s number talk, for example, is more strongly related to how often parents use number talk prompts to encourage their child’s engagement (e.g., questions about numerical concepts) than it is to how often parents make number talk statements [40]. Interestingly, the likelihood of prompting number talk (versus using statements about numbers) does not appear to vary by SES [40], but the field could use a fuller accounting of SES variations (or the lack thereof) in the quality and diversity of parent-child numerical talk.

Number talk, and math talk more generally, are also only one aspect of the early home learning environment that has been shown to differ as a function of SES, with many studies documenting variations in parent beliefs about learning, availability of learning resources (e.g., books, puzzles, games), caregiving supports (e.g., consistency and predictability), and the physical environment (e.g., lighting, crowding, noise; [25,28,59]). With math achievement gaps at kindergarten entry of more than 125% of a standard deviation separating children in the top 20% of the SES distribution from those in the bottom 20% in nationally representative data [53], it is likely that no one factor, alone—frequency of number talk or others—accounts for these differences. On the other hand, in contexts of such disadvantage, even small differences in math learning support—frequency of number talk or others—may be of great practical significance. Studies of relations between parent number talk and children’s math skills that have also included measures of general parent support of cognitive development (e.g., general language and learning stimulation during interactions) indicate complementary and unique links from SES to both numericalspecific talk and general learning support and in turn to math skills, thereby potentially compounding math learning advantages or disadvantages as a function of SES [7,15].

To this point, we have argued that small differences in the frequency of number talk may have important consequences for children’s math learning. Yet, the fact that parent education and family income only explain a very small portion of the variability observed in parent number talk is quite interesting. In other words, the distributions are largely overlapping, with relatively frequent number talk being a strength for many parents with low education and/or low income. Indeed, using the Rosenthal and Rubin [60] binomial approach to effect size interpretation, an *r* = 0.14 for income indicates that 43% of parents who were below the median on income were nonetheless above the median on number talk. Conversely, 43% of families who were above the median for income were below the median on number talk. This points to the critical importance of not overstating between-group differences and maintaining a strengths-based perspective regarding families in low SES contexts. The large overlap in number talk frequency across SES also evokes questions.

What household and/or parent factors may explain the remaining differences? Why do education and income not explain more of the variance? One avenue of speculation we see as worth exploring follows from the reality that parents’ work lives can be numerically (and mathematically, more generally) rich across the socioeconomic spectrum, whether it be in professions such as architecture versus construction, sales management versus sales clerking, or automobile design versus auto mechanics. Moreover, across the SES spectrum, family life—hobbies, daily activities, family routines, and leisure time—may be more or less numerically enriched; variations in these domains may translate into greater or fewer opportunities to develop habits of, and preferences for, numerical talk with children. Furthermore, we have good evidence that math attitudes [61] and parents’ math skills matter [37]. Beyond these factors, it is also possible that links between parent education and family income may be stronger in some contexts, under some conditions, or for some families more than others. A secondary purpose of the present study was, in fact, to provide a preliminary exploration of potential moderators.

### 4.2. Exploring Moderators

Although we did not have the power (in terms of the number of individual studies) to conduct formal hypothesis testing of moderators, we explored several possible factors that could alter the size of the association between SES and parent number talk, including characteristics of the study designs and characteristics of the parents and children who were sampled. Despite the limited number of studies, it is noteworthy that 13 of the 15 potential moderators were correlated with study effect sizes (for the association between number talk and either parent education or family income) at *r* < 0.20, more than half of the correlations were *r* < 0.10, and in the exceptional cases when *r* was 0.25 or larger the pattern did not replicate for both education and income. The largest correlation emerged for child age as a negative predictor of the association between family income and parent number talk; effect sizes appeared smallest for studies with the oldest children.

Overall, however, there was very little evidence that effect sizes varied in a systematic way, an interesting finding in light of limited evidence of moderator effects in meta-analyses of SES and total parent talk [24]. Nonetheless, there is some evidence that correlations between SES and number talk may be larger when parents and children interact with non-math-related materials compared with math-related materials [33]. More generally, understanding the contexts and conditions under which SES correlations with number talk may be exacerbated or diminished is a critical area of study for informing intervention efforts. Importantly, regardless of effect size, our study cannot inform why SES may be related to number talk (e.g., we cannot disentangle child evocative effects from contextual effects), another critical area for future study.

### 4.3. Study Limitations

While our study provides the first attempt at summarizing mixed evidence on relations between SES and parent number talk, several limitations are noteworthy. First, meta-analyses are entirely reliant on the quality of the original studies included, and despite many strengths of these studies (e.g., rich observational data, validated parent talk data via correlations with child talk and child math outcomes, and a diverse range of families sampled) several, by design, had somewhat restricted ranges on the SES indicators of interest here. While we did not find much preliminary evidence that the SES range had an impact, it is possible that a wider range of parent education levels and family income levels within the individual studies could produce larger average correlations with family number talk across the studies.

Second, most of these studies rely on relatively brief interactions, in part because of the arduous task of coding lengthy observations. Given the relative infrequency of parent number talk, longer interactions may reveal different patterns of association than we report here, either stronger or weaker. We do see it as a strength, however, that where parent-child interactions were observed varied across studies (which was not correlated with effect size), helping rule out the possibility that SES differences may be specific to either home or lab settings. Yet, the third notable limitation of the present study was that we were not able to examine all variations across studies (given the limited number of studies and some inconsistencies in the field) in the manner in which parent number talk data were coded. It remains possible that certain types of number talk may vary to a greater or lesser extent by SES than others, with reasonable suspects being whether researchers focus on overall number talk or sets of subdomains (and precisely which subdomains).

Fourth, while our exploratory moderator analyses address some of the between-study differences in design, other methodological factors that we could not examine—variations for which categorizing studies according to design would lead to just one or two studies in categories—may impact the size of the correlation between SES and number talk [18]. As this literature continues to grow, methodological factors worth considering (beyond those we directly address) include the types of activities being observed (e.g., unguided play, family routines, games) and the types of number talk being studied. As noted, for example, Thippana and colleagues [33] found that correlations between number talk and parent education were evident only during activities that were not explicitly math-related, with no such correlation in math-related activities. Given that most studies included in this meta-analysis used interaction materials provided by researchers (i.e., materials believed to provide chances for observing number talk), we cannot be certain that our results generalize to the materials families typically choose to play with (or choose not to play with) in their homes and communities.

Moreover, while we collapsed across studies with domain-general measures of number talk and those that focus on specific domains (e.g., labeling set sizes), it would be valuable for future work to disentangle the two. Precisely how number talk is coded may also matter; for example, whether researchers consider every number word in a count series as a distinct instance of number talk versus considering the entire series as one instance of number talk may impact the size of SES differences. It is also critical to recognize that our consideration of the racial and ethnic makeup of the samples as a moderator was only a starting point; because we focused only on pan-racial/ethnic groups—and the extent to which studies included minoritized groups—much greater nuance and specificity is needed as the literature grows. On a similar note, only one study in our meta-analysis came from outside the United States, and nearly all were focused on mother-child dyadic interactions.

Finally, it is worth a reminder that our moderator analyses are not adequately powered. This is not an uncommon limit in meta-analyses (which are often based on as few as eight studies), but it is an important one [50,51]. With so few studies, our moderator analyses are purely exploratory and deserve more attention as this literature base grows. Despite these limitations, however, we see considerable value in providing the first summative evaluation of links between SES and parent number talk.

## 5. Conclusions

Understanding whether the frequency of parent number talk varies by family socioeconomic conditions is critical for informing the study of early math learning in that it helps answer the question: why does parent number talk vary across families? In turn, the answer to this question may inform intervention efforts, a valuable line of inquiry in its own right given evidence of links between parent number talk in early childhood and children’s later math skills. In this respect, there is considerable value in continued work examining other factors, beyond education and income, which may be contributing to variation in parent math talk, including factors such as attitudes, math anxiety, occupations, and other contextual or social variations in family life. The present study helps build toward a fuller understanding of between-family differences, but somewhat provocatively so: parent education and family income are neither unrelated nor strongly related to parent number talk, despite very large disparities in children’s numerical skills. Modest associations may belie cumulative impacts of parent numerical talk across early childhood that advantage children in higher SES homes, but much more evidence is needed as to whether this is in fact the case. Indeed, our study also points to considerable variability in the frequency of number talk among families in both lower and higher SES contexts, and the fact that number talk frequency distributions for these groups overlap considerably.

## Figures and Tables

**Table 1.** Study Sample Sizes and Sample Demographic Characteristics.

Study	*n*	Age in Months *M* (*SD*)	Proportion Families of Color (Racial/Ethnic Category Most Represented)	Parent Education, yrs *M* (*SD*)	Family Income *M* (*SD*)
Casey et al., 2020	84	79 (4.2)	36% (11% multiracial)	17.09 (1.99)	$127,636 (68,374)
Eason & Ramani, 2020	69	57.6 (5.2)	35% (29% multiracial)	17.4 (1.76)	Not collected
Eason et al., 2021 ^a^	50	42.8 (10.6)	24.5% (9% Latinx)	15.88 (1.83)	Not collected
Eliott, Braham, & Libertus, 2017	44	69.6 (7.6)	11% (7% Black/African American)	15.64 (0.81)	Not collected
Gunderson & Levine, 2011	44	22 (14–30) ^b^	29.5% (13.6% African American)	15.90 (2.10)	$61,818 (31,542)
Laski & Collins, 2013	44	75 (6.96)	41% (16% Black/African American)	15.64 (2.41)	$87,525 (55,575)
Lombardi & Dearing, 2021	119	36	15.0% (5.0% Black; 5.0% Latinx)	14.17 (2.62)	$90,784 (64,741)
Lu et al. 2022	73	61.6 (5.96)	100% (Mandarin-speaking Chinese)	15.91 (1.51)	$74,378 ^b^ (49,496)
Nelson et al., 2019	28	49.4 (9.2)	82.1% (53.5% Black/African American)	16.11 (1.75)	$78,462 (28,662)
Ramani et al., 2015	33	52	91% (67% Black/African American)	13.15 (1.52)	$26,875 (13,060)
Susperreguy & Davis-Kean, 2016	33	54 (5.5)	35% (20% African American)	15.50 (2.17)	$72,349 (43,451)
Thippana et al., 2020	95 ^c^	47 (0.79)	15% (5% Black/African American; 5% Hispanic/Latino)	15.56 (1.80)	$101,333 (52,514)

a See also Chan et al., 2020 and Clements et al., 2021 for additional studies conducted with the same sample [42,43].

b Longitudinal assessments of parent numerical talk at child ages 14, 18, 22, 26, and 30 months.

c Sample size varied by observation location and SES variable (i.e., 95 for education and lab visit, 88 for income and lab visit, 87 for education and home video call, 80 for income and home video call).

**Table 2.** Numerical Talk Measures, Observation Sites, and Study Interaction Tasks.

Study	Measure of Numerical Talk	Observation Site & Task
Casey et al., 2020	Frequency of math facts hints about numbers on cards and decomposition	Home (semi-structured)
Eason & Ramani, 2020	Frequency of talk about numbers	Lab (structured, guided play, and unguided play)
Eason et al., 2021	Frequency of talk about counting, cardinality, equal distribution, fraction, magnitude, ordinality	Community site (semi-structured)
Eliott, Braham, & Libertus, 2017	Frequency of number words	Lab (semi-structured)
Gunderson & Levine, 2011	Frequency of talk about numbers	Home (daily activities; unstructured)
Laski & Collins, 2013	Frequency of numerical concept utterances	Lab or community site (semi-structured, random assignment to board game conditions)
Lombardi & Dearing, 2021	Ratings (1–3) of quality/quantity of labeling set size support	Lab (semi-structured)
Lu et al., 2022	Frequency of talk about counting, number labeling, cardinality, equality, quantifying without numbers, specific comparison, addition/subtraction, multiplication/division, and other advanced numerical talks.	Home (semi-structured, randomly assigned toy sets)
Nelson et al., 2019	Frequency of talk about cardinality, counting objects, arithmetic, magnitude comparisons	Home (cooking; semi-structured, random assignment to cookbook conditions)
Ramani et al., 2015	Frequency of talk about ordinal relations, cardinality, arithmetic	Head Start site (semi-structured)
Susperreguy & Davis-Kean, 2016	Frequency of talk about cardinality, counting, naming digits, units of measure, conventional nominatives, and number comparisons	Home (mealtime; unstructured)
Thippana et al., 2020	Frequency of number words	Home (unstructured free play) and Lab (semi-structured)

## Data Availability

All correlations and standard errors as well as moderator codes are available from the first author upon request.
